# Supplementary material for: Effective Perturbations on the Amplitude and Hysteresis of Erg-Mediated Potassium Current Caused by 1-Octylnonyl 8-[(2-hydroxyethyl)[6-oxo-6(undecyloxy)hexyl]amino]-octanoate (SM-102), a Cationic Lipid
Source: Biomedicines. 2021 Oct 1;9(10):1367. doi: 10.3390/biomedicines9101367 (PMC8533363; doi:10.3390/biomedicines9101367)
Supplement: Supplementary file 1 [file biomedicines-09-01367-s001.zip › biomedicines-1375471-supplementary.pdf]

Supplementary Information regarding chemical structure of SM-102, spermine, and glyceryl nonivamide (GLNVA) used in this study

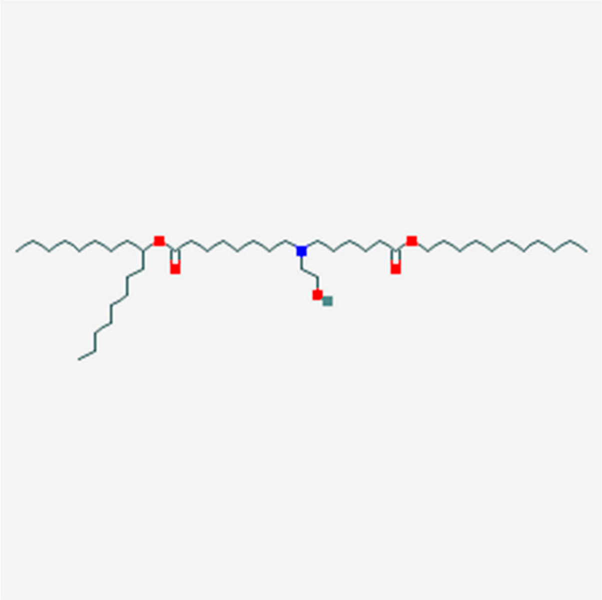

Figure S1. Chemical structure for SM-102 (heptadecan-9-yl 8-((2-hydroxyethyl)(6-oxo-6-(undecyloxyl)hexyl)amino)octanoate, 4-hydroxybutyl)azanediyl)bis(hexane-6,1-diyl)bis(2-hexyldecanoate, 1-octylnonyl 8-[(2-hydroxyethyl)[6-oxo-6-(undecyloxy)hexyl]amino]-octanoate). The structure was originally derived from https://pubchem.ncbi.nlm.nih.gov/compound/126697616.

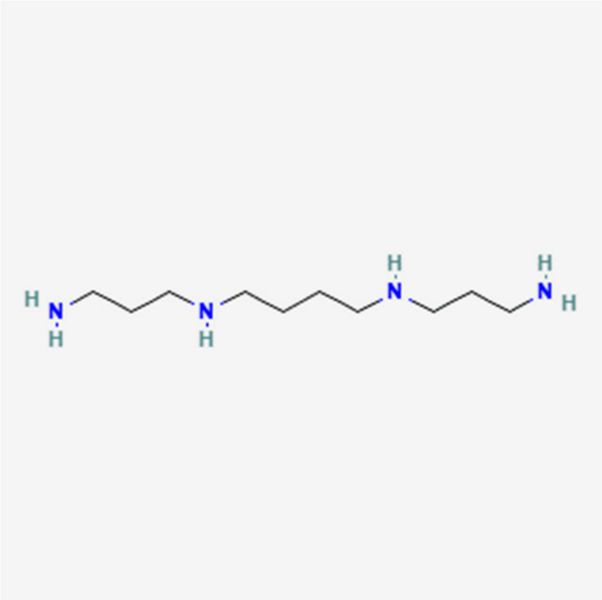

Figure S2. Chemical structure for spermine (N,N′-bis(3-aminopropyl)-1,4-diaminobutane). The structure was originally derived from https://pubchem.ncbi.nlm.nih.gov/compound/126697616.https://pubchem.ncbi.nlm.nih.gov/compound/1103.

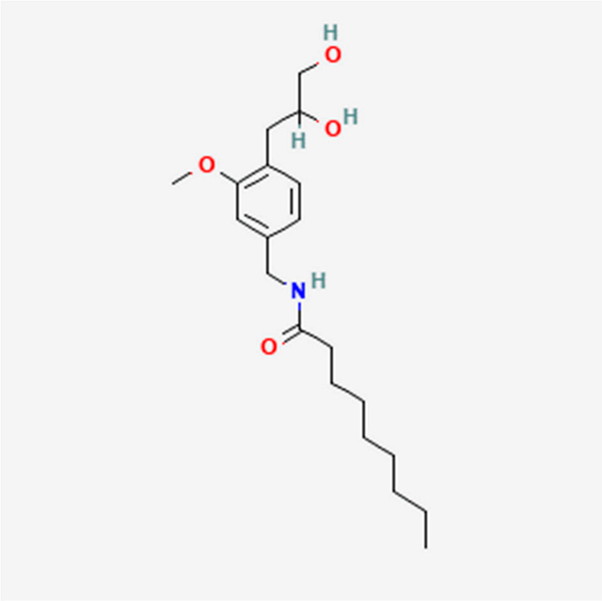

Figure S3. Chemical structure of glyceryl nonivamde (GLNVA). The structure was originally derived from https://pubchem.ncbi.nlm.nih.gov/compound/3035784.
